# Promising pharmacological profile of a Kunitz-type inhibitor in murine renal cell carcinoma model

**DOI:** 10.18632/oncotarget.11555

**Published:** 2016-08-23

**Authors:** Jean Gabriel de Souza, Katia L.P. Morais, Eduardo Anglés-Cano, Pamela Boufleur, Evandro Sobroza de Mello, Durvanei Augusto Maria, Clarice Silvia Taemi Origassa, Hamilton de Campos Zampolli, Niels Olsen Saraiva Câmara, Carolina Maria Berra, Rosemary Viola Bosch, Ana Marisa Chudzinski-Tavassi

**Affiliations:** ^1^ Biochemistry and Biophysics Laboratory, Butantan Institute, SP, Brazil; ^2^ Department of Biochemistry, Federal University of São Paulo, SP, Brazil; ^3^ CENTD- Center of Excellence in New Target Discovery, Butantan Institute, SP, Brazil; ^4^ INSERM UMR_S 1140-Université Paris Descartes, Sorbonne Paris Cité, Paris, France; ^5^ Department of Pathology, University of Sao Paulo Medical School, SP, Brazil; ^6^ Laboratory of Transplantation Immunobiology, Department of Immunology, Institute of Biomedical Sciences IV, University of São Paulo, SP, Brazil; ^7^ Division of Urology, Arnaldo Vieira de Carvalho Cancer Institute, SP, Brazil; ^8^ Nephrology Division, Federal University of São Paulo, SP, Brazil

**Keywords:** renal cell carcinoma, amblyomin-X, antitumor activity, tumor resistance, tumor affinity

## Abstract

Renal cell carcinoma (RCC), also called kidney cancer or renal adenocarcinoma, is highly resistant to current treatments. It has been previously reported that a Kunitz-type inhibitor domain-containing protein, isolated from the salivary glands of the *Amblyomma cajennense* tick, triggers apoptosis in murine renal adenocarcinoma cells (Renca) by inhibiting the proteasome and endoplasmic reticulum stress. Of note, Amblyomin-X is the corresponding recombinant protein identified in the cDNA library from *A. cajennense* salivary glands. Herein, using orthotopic kidney tumors in mice, we demonstrate that Amblyomin-X is able to drastically reduce the incidence of lung metastases by inducing cell cycle arrest and apoptosis. The *in vitro* assays show that Amblyomin-X is capable of reducing the proliferation rate of Renca cells, promoting cell cycle arrest, and down-regulating the expression of crucial proteins (cyclin D1, Ki67 and Pgp) involved in the aggressiveness and resistance of RCC. Regarding non-tumor cells (NIH3T3), Amblyomin-X produced minor effects in the cyclin D1 levels. Interestingly, observing the image assays, the fluorescence-labelled Amblyomin-X was indeed detected in the tumor stroma whereas in healthy animals it was rapidly metabolized and excreted. Taken the findings together, Amblyomin-X can be considered as a potential anti-RCC drug candidate.

## INTRODUCTION

Renal cell carcinoma (RCC) is responsible for approximately 2% of malignancies that affect adults and for 90–95% of all kidney cancers [1, 2]. RCC secretes proangiogenic cytokines, leading to the activation of the PI3K/Akt/mTOR signaling pathway and the overexpression of the proliferative protein Ki-67 [3–6]. In this regard, the available treatments for RCC are a combining targeted therapy that includes anti-angiogenic agents (tyrosine kinase inhibitors, such as sorafenib, sunitinib, pazopanib, and axitinib), or an anti-angiogenic antibody routinely employed in combination with interferon alpha (bevacizumab) [7, 8], and antiproliferative agents (mTOR inhibitors, such as temsirolimus and everolimus) [9]. However, the effectiveness of these therapies are associated with high toxicity and, consequently, serious adverse events, and the progression-free survival (PFS) measured is limited to two years [10]. In the setting of localized RCC disease, surgery remains the mainstay of a good response [11]. Nonetheless, RCC has been considered as “immunogenic” cancer [12], since some spontaneous remission eventually can occur suggesting that the immune system may play an important role in disease treatment [13]. In this context, clinical trials involving immunotherapy based on cytokines, such as interleukin 2 (IL-2) and/or interferon-alpha (IFN-α), and also employing monoclonal antibodies, such as nivolumab (anti-PD-1) and impilimumab (anti-CTLA4), against immune check-points, have been shown to enhance the antitumor immunity [14]. Another complication regarding the RCC treatment is related to the tumor cells genes overexpression, such as P-glycoprotein (Pgp), which can confer multidrug resistance [15]. Thus, the discovery of drug candidates that could be more effective for treating this kind of disease is an urgent need.

Amblyomin-X (GenBank code: AAT68575), a recombinant Kunitz-type protease inhibitor protein (12.4 kDa) identified in the cDNA library from salivary glands of the *Amblyomma cajennense* tick, has shown to induce pro-apoptotic effects in different tumor cell lines including murine renal adenocarnoma cells (Renca) [16, 17]. However, in Renca cells only a small percent of the cells upon Amblyomin-X treatment are positive to propidium iodide (necrosis) [17]. In these *in vitro* studies, Amblyomin-X was able to induce the activation of the intrinsic apoptosis pathway (increase of cytochrome-c and caspase-3, and reduction of Bcl-2 expression) through proteasome inhibition and endoplasmic reticulum (ER) stress [18, 19]. In a mouse melanoma model (primary tumor), Amblyomin-X has shown antitumor activity and an antithrombotic effect [20, 21]. Furthermore, it inhibits the angiogenic capacities of the t-End endothelial cell line *in vitro* [22] and produces an anti-angiogenic effect *in vivo* in the dorsal skinfold chamber and chick embryo chorioallantoic membrane (CAM) assays [23].

Interestingly, the lack of suffering observed in t-End cells as well as in murine and human fibroblasts treated with Amblyomin-X may also support its tumor cell selectivity [18, 19].

We have investigated, herein, the Amblyomin-X pharmacology safety through an acute toxicity assay using healthy mice. In addition, regarding animals orthotopically implanted with renal cell carcinoma, the protein's tumor affinity and biodistribution were also assessed by employing *in vivo* image approach. Moreover, *in vitro* assays were performed using Renca and non-tumor (NIH3T3) cells to measure the biomarkers protein-levels in cell proliferation, apoptosis-cell death, and multi-drug resistance, which have been reported as crucial points to RCC therapeutic.

Summing up, our findings show that Amblyomin-X can be rapidly eliminated by healthy animal systems, and display antitumor activity in a selective fashion. Also, the cyclin D1, Ki67, and Pgp protein levels were down-regulated in Renca cells. Of note, a minor intensity reduction only in cyclin D1 was observed in NIH3T3 cells. Regarding Renca cells, Amblyomin-X has promoted cell cycle arrest and apoptosis, which was reinforced by the Bcl-2 reduction in the orthotopic kidney tumor model.

## RESULTS

### Tissues of healthy animals remained preserved after Amblyomin-X treatment

**Figure 1:**
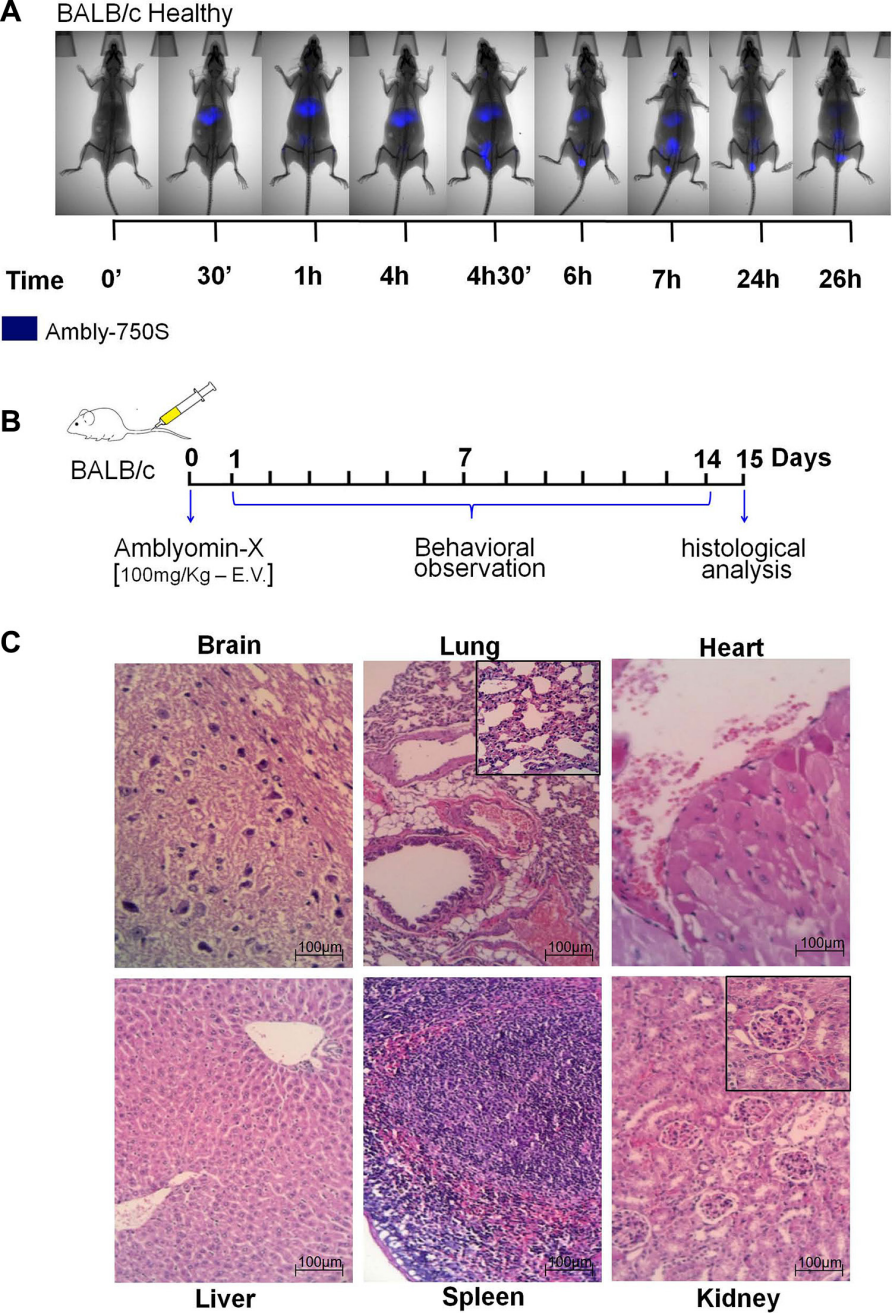
Acute toxicity assay The Amblyomin-X acute toxicity was carried out in healthy male BALB/c mice. (**A**) Animals were treated with a single dose of Ambly750S (1 mg/Kg) and the distribution of the labeled compound was monitored at different time points, using a CareStream instrument. (**B**) Animals were treated with a single dose of Amblyomin-X (100 mg/Kg), which corresponds to more than 100-fold the effective dose, and after 14 days the organs were collected and processed for (**C**) histological analyses.

In healthy BALB/c mice, Ambly750S appeared in the liver 30 min after administration. Its renal excretion started 4 h later though (see Figure 1A). Regarding 26 h, there was only a fluorescence trace in the renal region and in the urinary tract. Likewise, in healthy BALB/c mice treated with Amblyomin-X, using more than 100-fold the effective dose, no behavior alterations, such as vocalization, piloerection, coordination disorder or alimentary disruptions (Figure 1B), were observed. In addition, the brain, lung, heart, liver, spleen, and kidney tissues had no histological lesions detected (Figure 1C).

### Amblyomin-X reduces the renca cell proliferation rate by cycle arrest

**Figure 2:**
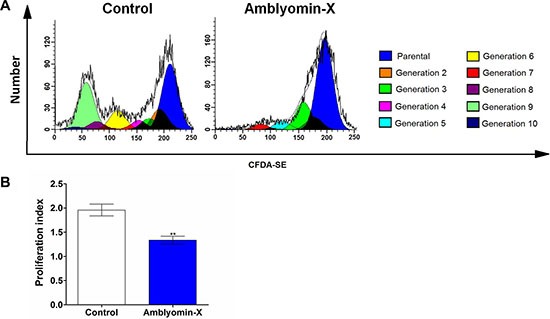
Antiproliferative effects of Amblyomin-X on Renca cells Renca cells were seeded in six-well plates (2 × 10^5^ cells/well), incubated with 5 μM CFDA-SE for 15 min and, then, treated with Amblyomin-X (0.5 μM) for 24 h. The cellular CFDA-SE fluorescence intensity was estimated by flow cytometry. (**A**) Histograms considering each daughter cells generation, different color peaks indicated generations of daughter cells. (**B**) Bar plots of proliferation index regarding parental cells was calculated by using a software (WinMDI Version 2.8), regarding three independent experiments. *p ≤ 0.05, **p ≤ 0.005 compared to the control, as determined by unpaired *t*-tests with Welch's correction.

Amblyomin-X inhibited cell proliferation after 24 h. As demonstrated in the Figure 2A, different color peaks have indicated different generations of daughter cells distribution, determining up to 7–10 cell cycles of division in the Renca cells (Amblyomin-X treatment – untreated control, respectively). The shift in the fluorescence signal in the Renca-treated parental cells appointed a reduction in the proliferation index compared to untreated control (Figure 2B). The effect of Amblyomin-X in the phases of the cell cycle was also investigated; the anti proliferative and/or pro-apoptotic drug effects promoted cell cycle alterations, triggering a reduction in the cell proliferation rate. In Renca cells, the Amblyomin-X treatment for 24 h increased the percentage of cells in G0/G1 phase, and decreased the percentage of cells in S or G2/M phase compared to the untreated cells (Table 1). In agreement with previous studies, sub-diploid cells were observed, indicating apoptosis. In contrast, fibroblasts (NIH3T3) were preserved, and insignificant alterations were observed in G1 phase of cell cycle, reinforcing the selectivity of Amblyomin-X on tumor cells.

**Table 1:** Cell cycle analysis of Renca and NIH3T3 cells treated with Amblyomin-X

Cell cycle arrest (24 h)				
Cells	Treatment	Cells in each phase of the cell cycle (%)		
		G0/G1	S	G2/M
Renca	Untreated control	58 ± 2	10 ± 2	26 ± 3
	0.5 μM Amblyomin-X	76 ± 2**	5 ± 1*	4 ± 1**
NIH3T3	Untreated control	59 ± 2	11 ± 1	27 ± 3
	0.5 μM Amblyomin-X	67 ± 1	8 ± 1	20 ± 1

Renca and NIH3T3 cells were incubated with 0.5 μM Amblyomin-X for 24 h, and cell cycle distribution was assessed by flow cytometry. The data are presented as the mean ± SD of three independent experiments.

* *p* ≤ 0.05,

** *p* ≤ 0.005 compared to the untreated control group, as determined by unpaired *t*-test with welch's correction.

### Modulation of biomarkers on survival and proliferation

**Figure 3:**
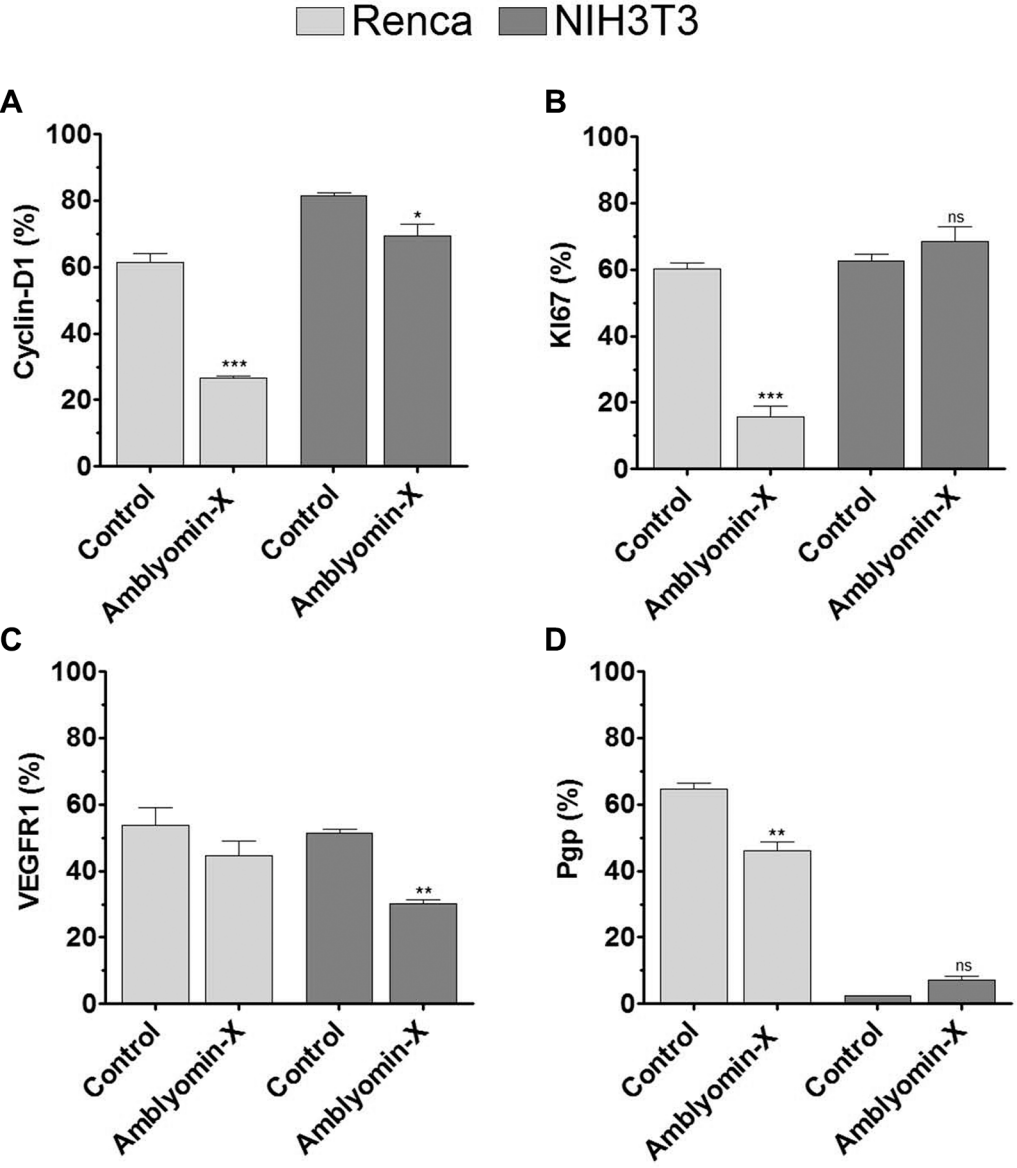
Amblyomin-X affects RCC biomarkers Renca and NIH3T3 cells were treated with Amblyomin-X (0.1 μM) for 24 h. Then, the cells were collected, permeabilized, and incubated with appropriate antibodies as described in Materials and Methods. (**A**) Cyclin D1. (**B**) Ki67. (**C**) VEGFR1. (**D**) Pgp. The experiments were performed using flow cytometry. The data are presented as the mean ± SEM. *p ≤ 0.05, **p ≤ 0.005 compared to the control, as determined by unpaired *t*-tests with Welch's correction. The ns abbreviation stands for not significant.

Consistent with the cell proliferation and cell cycle findings, the Amblyomin-X treatment has changed the protein levels involved on survival and proliferation. In Renca cells the levels of cyclin D1 and Ki67, two cell proliferation markers, decreased by 50% and 75%, respectively (Figure 3A and 3B). However, the VEGFR1 expression levels did not change (Figure 3C). The Pgp levels (an efflux pump protein that confers resistance to multiple drugs by reducing the intracellular concentration of cytotoxic agents) decreased by approximately 15% (Figure 3D). In order to determine whether the effects of Amblyomin-X are selective on tumor cells, the same biomarkers were investigated in NIH3T3 (non-tumor) cells. Of note, only the cyclin D1 and VEGFR1 expression levels were decreased, around 15% and 40%, respectively, after the treatment with Amblyomin-X (Figure 3A and 3C).

### Avidity of Amblyomin-X for renal tumor mass

**Figure 4:**
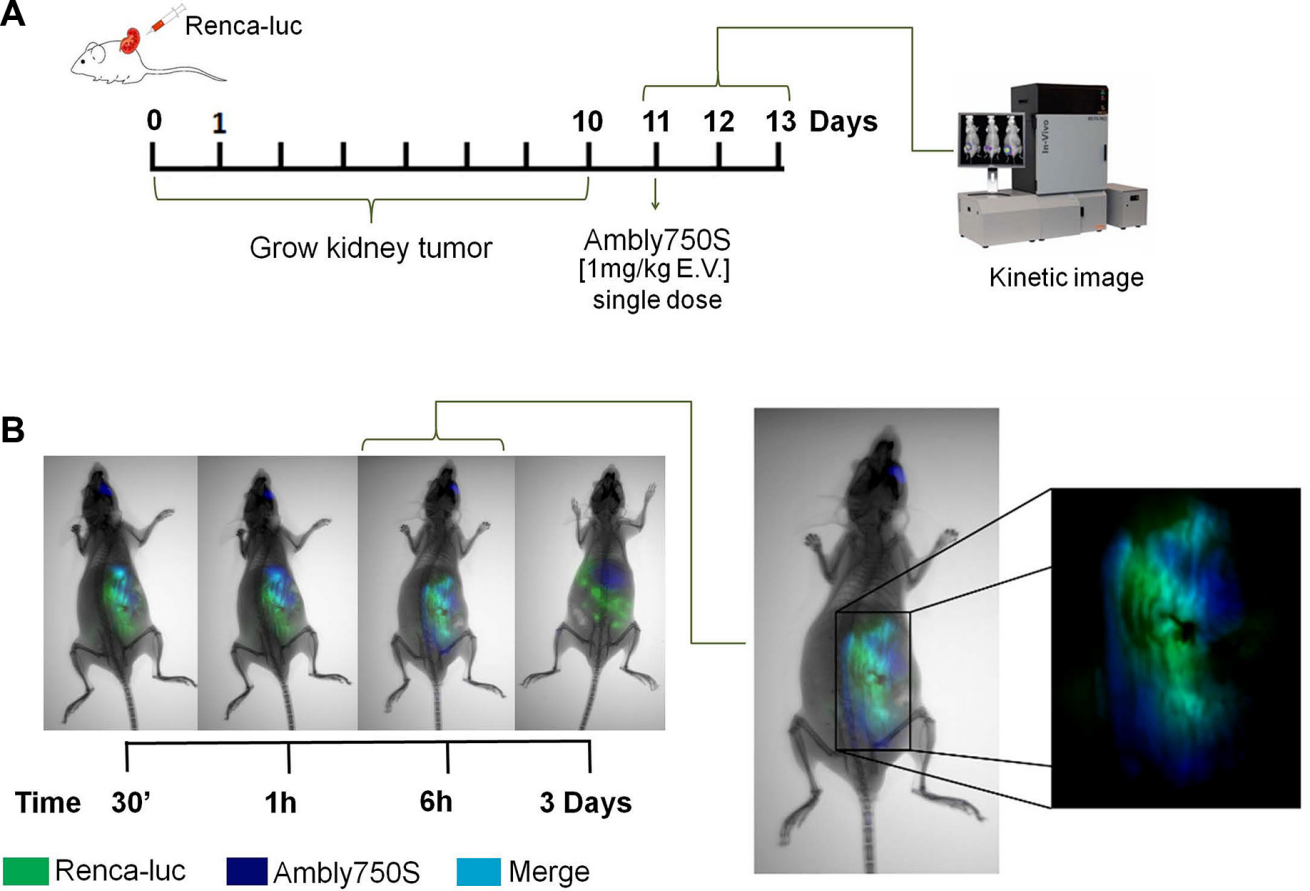
Co-localization of Amblyomin-X on tumor or tumor stroma The procedure was carried out with animals bearing RCC. (**A**) After 10 days of renal implant, the animals with the tumors derived from luciferase stably transfected Renca cells (Renca-luc) were imaged after luciferin injection and Ambly750S (1 mg/Kg) administration, using a CareStream instrument. (**B**) Representative images for the X-ray, luminescence, and fluorescence procedures. Luminescence identified by the green color points out the tumor regions. Fluorescence (EM750 nm EX790 nm) identified by the blue color indicates the location of Ambly750S. The cyan color indicates co-localization, consisting of green (Renca-luc) and blue (Ambly750S) colors.

In order to determine the Ambly750s avidity for Renca tumor mass, the orthotopic kidney tumor model was developed using luciferase-expressing Renca cells (Renca-luc). At the 11^th^ day, a single dose of Ambly750s (1 mg/Kg) was intravenously administered, and the *in vivo* image assay was carried out (Figure 4A). Clearly, Ambly750S was localized in the tumor stroma after 30 min of administration, and has remained there for 3 days (Figure 4B). It is noteworthy that the purpose of the single dose was to evaluate the compound's tumor avidity, not the therapeutic effect.

### Antimetastatic activity of Amblyomin-X in an orthotopic tumor model

**Figure 5:**
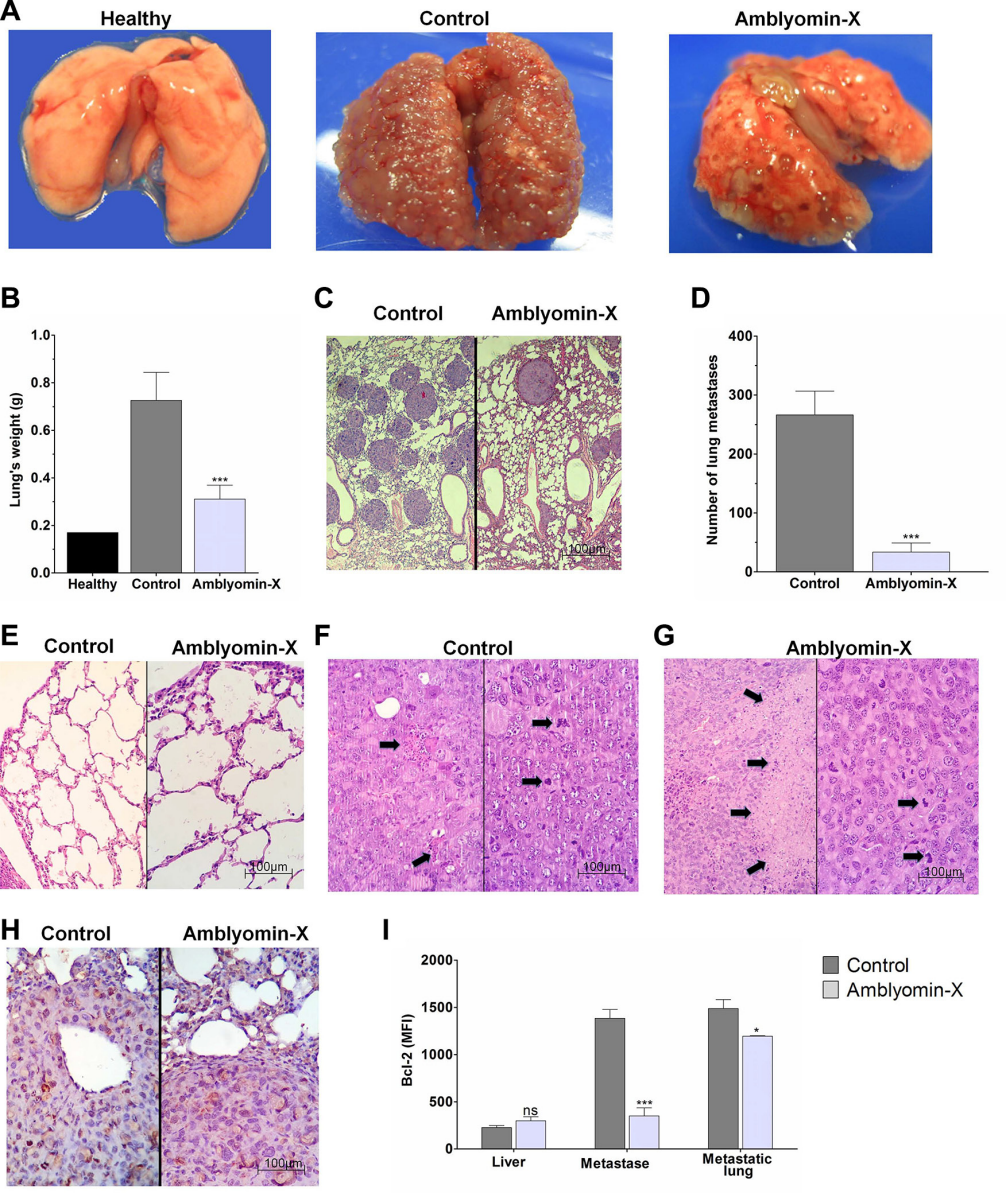
Amblyomin-X antitumor effectiveness in a mouse renal orthotopic model Renca cells (1 × 10^6^ cells/mouse) were injected into the renal subcapsule of BALB/c mice. Ten days later the kidney with the primary tumor was surgically removed and 24 h later each mouse received saline or Amblyomin-X (1 mg/kg) daily for 14 days. (**A**) Representative pictures of the mouse lungs at the end of treatment. (**B**) Bar plot representing the mean ± SD of lung weights per group of five mice in three independent assays. (**C**–**G**) Histological analysis: (C) Untreated (control; left) and treated (Amblyomin-X; right) mice lung images showing a reduction of the number of metastases (D) Bar plot representing the number of metastases reduction. (E) Untreated (control; left) and treated (Amblyomin-X; right) lungs showing non-tumor parenchyma with histologically normal appearance. (F) Control untreated mice presented high mitotic index (right) accompanied by scarce necrotic foci (left), representing only 5% of the tumor section area (arrows); (G) Amblyomin-X treated mice presented high mitotic index (right) accompanied by extensive necrotic areas (left), representing 30% of the section area (arrows). (**H**) Ki67 expression evaluation by immunohistochemistry analyses of the lung metastases. Ki-67 assessment was obtained by using light microscopy in non-selected five different tumor areas, counting at least 1000 nuclei. Light stained nuclei were also considered positive. (**I**) Bcl-2 expression in the liver, peritoneal metastases and metastatic lungs, expressed by intensity mean of fluorescence. The values were acquired using flow cytometry and the data are presented as the mean ± SEM (three independents assays containing *N* = 4 per group). *p ≤ 0.05, ***p ≤ 0.0001 compared to respective control, as determined by unpaired *t-test* with welch's correction. The ns abbreviation stands for not significant.

*In vivo* experimentation has demonstrated that the Amblyomin-X treatment drastically reduces the incidence of lung metastases in comparison to the saline treatment (Figure 5A–5D). The lungs weight decreased 57 % in comparison to the control (see Figure 5B). In addition, a reduction of the number of metastases (87%) was observed (Figure 5D), whereas the lungs parenchyma of the same animals was unaffected by the treatment (Figure 5E). Histological analysis of the lungs revealed 5% tumor necrosis in the control specimens (Figure 5F) whereas 30% tumor necrosis was observed in the treated animals (Figure 5G). The mitotic indexes were extremely high in both untreated (control) and treated animal tumors. Metastatic lung Ki67 immunohistochemistry revealed about 30% of brown-stained positive nuclei in control animals whereas only 5–7% of cells showed positivity to Ki-67 in metastatic lungs of treated animals (Figure 5H). Although no difference between control and treated groups was observed for Bcl-2 levels measured in metastasis-free liver, in metastasis isolated from the peritoneal cavity, Bcl-2 protein was down-regulated to 75% in the Amblyomin-X-treated group. However, in the homogenate of metastatic lungs, which has a significant portion of healthy tissue, the reduction for the treated group was 20% only (Figure 5I).

## DISCUSSION

Despite the recent advances, the RCC treatment still remains as a challenge for oncologists. In RCC, primary tumors are well vascularized, and hematogenous metastases are found most often in the lungs, bones, and lymph nodes. The RCC treatment currently includes surgical management of localized renal cancer [11, 24], or antiproliferative and anti-angiogenic agents combined with immunotherapy or radiotherapy to metastatic disease. However, all these therapeutic alternatives have improved neither the prolongation of overall survival nor the health-related quality of life score (HRQoL) [25]. The most common adverse events related to the antiproliferative and anti-angiogenic current treatments are diarrhea, hypertension, hair color changes, nausea, anorexia, fatigue, and vomiting [8, 26]. These effects are associated with low selectivity-treatment, leading to cytotoxicity in different levels of aggressiveness depending on the drug.

In this study, when Ambly750S (1 mg/kg) was administered to healthy animals it was rapidly metabolized and mainly excreted by the kidneys (Figure 1A). In addition, in the histological analysis no tissues injuries were found even after an intravenous injection of Amblyomin-X 100-fold higher than the effective dose. Furthermore, side effects such as physiologic, alimentary or behavioral disorders were not observed.

Moreover, *in vitro* analyses on cell precursor's frequency and the cellular proliferation index indicated that the Amblyomin-X treatment reduced the Renca cells proliferation rate; this effect was accompanied by a cell cycle arrest. This cellular response could be related to the Amblyomin-X-induced proteasome inhibition and ER stress in Renca cells, previously reported by our group [18, 27]. The proteasome is important for modulating the activity of the cyclin-dependent inhibitors p21 and p27 [28]. Therefore, changes in its activity can lead to perturbations in the cell cycle. Also, Amblyomin-X-induced ER stress in Renca cells is accompanied by the induction of GADD153 expression, which can lead to cell cycle arrest [29].

Considering the findings, we evaluated the cell proliferation biomarkers in Renca cells and found that the Amblyomin-X treatment has decreased the protein levels of Ki67, which is a cell proliferation marker associated with a poor prognosis in RCC patients [6]. Interestingly, recent studies have shown that Ki67 knockdown leads to cell death in human renal carcinoma [30], highlighting the importance of drugs that regulate this protein. Moreover, the Amblyomin-X treatment has also decreased the cyclin D1 protein levels. Cyclin D1 is involved in the G1 to S phase transition and can be considered as an indicator for the proliferative response.

Although no changes in the VEGFR1 protein levels were observed in Renca cells, Drewes and colleagues have reported the Amblyomin-X anti-angiogenic effects [23]. In addition, they demonstrated post-transcriptional alterations in other processes related to angiogenesis, such as cytokine secretion and adhesion molecules in endothelial cells (integrins) involved in the Amblyomin-X mechanism of action as an antitumor agent [22].

In contrast to other drugs under study for treating RCC [15], Amblyomin-X decreases the Pgp protein levels in Renca cells. The ability to reverse multidrug resistance of chemotherapeutics has been widely investigated using verapamil and cyclosporine A, for instance [31]. However, the clinical use of Pgp inhibitors can be generally complicated due to both, the pharmacokinetic interactions resulting from inhibiting Pgp in normal tissues, increasing the toxicity associated with chemotherapy, and the modulation of the pharmacological effects of the therapeutic agent itself, which often produces severe toxicity at the levels required to effectively block the Pgp activity in tumors [32]. In this regard, we have proposed that Amblyomin-X is able to down regulate the cellular pathways which promote the Renca cell survival, and those pathways would be probably related to tumor maintenance and metastasis induction.

On the other hand, the evaluation of the same RCC biomarkers in non-tumoral NIH3T3 cells showed a discrete alteration in the cyclin D1 protein levels. That change should not be drastic, since the cell cycle has remained unaltered. The VEGFR1 protein levels decreased considerably in NIH3T3cells when treated with Amblyomin-X. However, NIH3T3 cells viability remained the same under treatment, whatever the biomarkers used in this study [18], or at the highest dose (data not shown). Thus, any perturbation caused by Amblyomin-X seems to be restored in non-tumor cells. Corroborating with previous findings, Amblyomin-X acts distinctively on tumor and non-tumor cells [18, 20].

Considering our previous results on the antitumor activity of Amblyomin-X on primary melanoma tumors (B16F10) [21] and the equal mechanisms of cell death related to Melanoma and Renca cells [18, 19, 21], we used the orthotopic renal cell carcinoma model to focuss the effect of Amblyomin-X on metastatic RCC. The kidney tumor model in mice mimics the disease and clinical process observed in humans, and it has been particularly suitable for assessing novel therapeutic approaches [12]. We used Ambly750S and Renca-luc to evaluate the Amblyomin-X affinity for the tumor (Figure 1). Notably, Amblyomin-X was detected in tumor stroma after 30 min, emphasizing its selectivity for tumor cells. Furthermore, comparing the excretion time between healthy animals (Figure 1A) and animals bearing RCC (Figure 4B), Amblyomin-X has remained three times upward in the tumor and was detected in the liver after 3 days. Thus, we have shown that the remarkable difference in Amblyomin-X cytotoxicity (*in vitro* and *in vivo*) on tumor and non-tumor cells would be associated with its preferential uptake by cancer cells.

Our findings have demonstrated that the Amblyomin-X monotherapy can significantly inhibit metastasis formation comparing untreated and treated animals. Moreover, the histological analyses have shown that the Amblyomin-X cytotoxicity was restricted to the tumor area. In addition, when the Bcl-2 anti-apoptotic protein-expression was measured in the liver, peritoneal metastases, and metastatic lungs, we observed a selective reduction. Therefore this selective reduction is in accordance with the previous results, that have shown imbalance between pro and anti-apoptotic Bcl-2 family proteins, including caspase-3 activated, in Renca cells treated Amblyomin-X, but not in non-tumor cells [18]. The findings also reinforce the selective anti-tumor effect regarding the *in vivo* treatment. However, the molecular mechanism involved in this kind of selectivity needs to be unveiled, and our research group is working to reach that goal. Preliminary results have pointed out to specific membrane interactions that may involve a membrane phospholipid, phosphatidylserine, which is translocated to the membrane outer leaflet in cancer cells. Amblyomin-X seems to be a safer potential drug candidate. Nowadays, the antineoplastic drugs for treating RCC have shown high cytotoxicity and tumor resistance, achieving better antitumor effects when used in combination with other therapeutic agents. In conclusion, Amblyomin-X exhibits selective antitumor activity in a renal cancer model. In renal cancer cells, Amblyomin-X triggers cellular responses, which act synergistically, and promotes down regulation of survival pathways that lead to cell death. Interestingly, Amblyomin-X exhibits a more driven or selective antitumor action emphasized by the reduced *in vivo* toxicity on non-tumor cells or tissues. Taken all findings together, Amblyomin-X could be considered as a potential anticancer drug candidate opening frontiers for future experimentation in anticancer therapy.

## MATERIALS AND METHODS

### Ethics statement

All experimental procedures were performed in accordance with the guidelines for animal experimentation approved by the Committee on Animal Research and Ethics (CARE) of Butantan Institute. The protocol used in this study was also approved by CARE (Permit Number: 994/12).

### Animals

The experiments were performed using male BALB/c mice (approximately 22 g each) bred in the animal care facility of Butantan Institute (Sao Paulo, SP, Brazil). All animals had free access to food and water, and they were subjected to a 12/12 h light/dark cycle. Ethical rules for animal care as outlined by the International Animal Welfare Recommendations and in accordance with the local institutional animal welfare guideline were followed. Surgical procedures were performed using anesthesia via intraperitoneal injection of ketamine-xylazine (10–2 mg/mL/kg) in combination with dipyrone administration (72 h pre- and post-operative). All efforts were taken into account in order to minimize suffering.

### Amblyomin-X obtention

Recombinant Amblyomin-X was produced using an *E. coli* expression system according to the methods developed at the Biochemistry and Biophysics Laboratory. The recombinant protein was expressed with no histidine tail or any other purification tag sequence. The host system used was an *E. coli* strain, BL21 DE3, which harbors a plasmid for the inducible (1 mM IPTG) expression of Amblyomin-X under the control of the T5 promoter. The antibiotic ampicillin was used for resistance selection. Inclusion bodies containing recombinant Amblyomin-X were harvested from high density cultures grown in a bioreactor and washed and solubilized under denaturing conditions. After refolding, soluble Amblyomin-X was captured and purified by ion exchange chromatography (Akta Pilot, GE Healthcare, Sweden). The eluted fractions were pooled and dialyzed by ultrafiltration using a 3 kDa cutoff (VersaFlux, GE Healthcare, Sweden). The purified protein was filtered through a sterile 0.22 μm membrane, and aliquotes were lyophilized and stored at −70°C until use.

The concentration was determined using a BCA assay (Thermo Scientific Pierce BCA Protein Assay) according to the manufacturer's instructions. Biochemical assays (SDS-PAGE, mass spectrometry, circular dichroism and MTT) were performed in order to confirm the integrity and activity of the protein, before further experiments.

### Cell lines and culture conditions

Murine renal adenocarcinoma cells (Renca) (No. CRL-2947, ATCC, Manassas, VA) were cultured in RPMI supplemented with 10% fetal bovine serum, 0.1 mM nonessential amino acids, 1 mM sodium pyruvate, 2 mM L-glutamine, 100 mg/mL streptomycin sulfate, and 100 U/mL penicillin G. NIH3T3 mouse fibroblast cells purchased from ATCC (n°. CRL-1658, Manassas, VA) were cultured according to the manufacturer's instructions. All cell lines were incubated in a humidified 5% CO_2_ incubator at 37°C.

### *In vivo* acute toxicity assay

Healthy BALB/c male mice were treated with Ambly750S (1 mg/Kg) intravenously into the orbital plexus. Then, the biodistribution of the labeled compound was followed at different times, using a CareStream instrument. An additional procedure was carried out where healthy BALB/c male mice were treated with 100 mg/Kg Amblyomin-X, which corresponds to more than one hundred-fold the effective dose used in the orthotopic kidney tumors model. After 14 days, the animals were euthanized by cardiac puncture under deep anesthesia. Then, the organs were collected and processed to perform histological analyses. Brain, lung, heart, liver, spleen and kidney tissues had H&E sections systematically searched for necrosis, inflammation, vascular damage or abnormal intracellular accumulations.

### Cell proliferation assay

Carboxyfluorescein diacetate succinimidyl ester (CFDA-SE) was used to evaluate the Renca cell proliferation. That compound is a lipophilic dye that reacts with the amino groups of peptides and proteins in order to form a stable amide bond. Also, it is equally partitioned among daughter cells during cell cycle division. According to the kit's manufacture, cell division results in sequential halving of CFDA-SE fluorescence resulting in a cellular fluorescence histogram in which the peaks represent parental generations. Renca cells were seeded in six-well plates (2 × 10^5^ cells/well), washed three times with PBS, and incubated with CFDA-SE (5 μM) in PBS, in the dark, at 37°C, in 5% CO_2_ for 15 min. After this period, the cells were treated with 0.5 μM Amblyomin-X for 24 h and were analyzed using a FACSCalibur™ (Becton Dickinson, USA) flow cytometer. The proliferation index about parental cells was calculated by using WinMDI Version 2.8 software.

### Cell cycle analysis

Regarding the DNA content analysis, Renca and NIH3T3 cells (1 × 10^6^) were treated with 0.1 μM Amblyomin-X for 24 h. Subsequently, the cells were trypsinized, washed with cold PBS, and fixed with 70% ethanol for approximately 16 h at 4°C. Then, the cells were washed three times with cold PBS and stained with propidium iodide (staining solution: 9.5 mL Triton X-100 0.1%, 0.2 mL RNase A 10 mg/mL, and 0.25 mL propidium iodide 1 mg/mL). The cells were examined using a FACSCalibur™ (Becton-Dickinson, USA) flow cytometer. The percentage of cells in each cell cycle phase was analyzed using FlowJo software.

### Determination of protein levels of RCC biomarkers

Renca and NIH3T3 cells (5 × 10^5^/well) were incubated with 0.1 μM Amblyomin-X for 24 h. Then, the cells were harvested, fixed in 4% paraformaldehyde in PBS and permeabilized with 0.5% saponin in FACS buffer (BD Biosciences). After that, the cells were incubated for 18 h, at 4°C, with the following mouse monoclonal antibodies at 1 μg/10^6^ cells concentration: anti-VEGFR1 or anti-Cyclin D1 (both from Santa Cruz Biotechnology) and anti-Ki67 MIB or anti-Pgp (both from BD Biosciences). After being washed with PBS, the cells were incubated with FITC-conjugated anti–mouse antibody. The cells were subjected to FACSCalibur™ (Becton Dickinson, USA) flow cytometry, and the data were analyzed with FlowJo software.

### Orthotopic kidney tumors model

The orthotopic tumor model was established by injecting 1×10^6^ Renca cells into the subcapsular space of the left kidney of BALB/c mice under anesthesia. At the 10th day, when the primary tumor had already developed, the animals were subjected to total nephrectomy. After 24 h, the animals were randomly split into 2 groups, each one composed by 8 animals, which received daily (during 14 days) an intraperitoneal injection of either 200 μL sterile saline or Amblyomin-X (1 mg/kg sterile saline). Then, the animals were euthanized by cardiac puncture under deep anesthesia, and the lungs were collected, weighed, and observed, in order to compare the number of metastases. Histological analyses were performed (i) to provide a quantitative analysis of lung metastasis; and, (ii) to inspect tumor and non-tumor lung parenchyma. Standard light microscopy immunohistochemistry analysis was conducted in five non-selected different tumor areas in order to evaluate cell proliferation, using an antibody against KI-67 (MIB-1, BD Biosciences) and counting at least 1000 nuclei. Light stained nuclei were also considered as positive result. All steps of this procedure were repeated three times, and can be found in Supplementary Figure S1.

### Determination of Bcl-2 protein in orthotopic kidney tumors model by flow cytometry

Flow cytometry was applied to evaluate the expression of an anti-apoptotic protein, Bcl-2 (Santa Cruz, USA) in the liver, peritoneal metastases, and metastatic lungs tissues, isolated from an orthotopic kidney tumor model, as previously mentioned. A combined mechanical and enzymatic approach for tissue disaggregation was applied. Briefly, the tissues were mechanically disrupted and submitted to an enzymatic digestion through colagenase type IV (Sigma) (2 mg/ml) incubation for 40 min at 37°C, under agitation, and neutralized with PBS-FBS 2.5%. The homogenate was filtered in the cell strainer 70 μm (Corning) and submitted to 1× RBC lysis buffer. Cells were adjusted to 1 × 10^6^ cells/mL and the labeling was performed according to manufacturer instructions, acquired by FACSCalibur™ (Becton Dickinson, USA) flow cytometry. The data were analyzed with FlowJo software.

### *In vivo* imaging of RCC tumors (model for tumor affinity)

Renca cells were stably transfected as described elsewhere [32]. Then, luciferase stably transfected Renca-luc cells (1 × 10^6^ cells) were orthotopically injected into the subcapsular space of the left kidney of BALB/c mice under anesthesia. For detection, Amblyomin-X was directly labeled with the amine-reactive dye using the Vivotag750S (Perkin Elmer^®^), following manufacturer instruction. The conjugated dye-protein was named Ambly750S.

At 11st day of tumor implant, the animals were anesthetized with isoflurane and intraperitoneally injected using 150 mg/kg D-luciferin (PerkinElmer, Ohio, USA). Simultaneously, a single Ambly750S dose (1 mg/Kg) was intravenously administered into the orbital plexus. Ten minutes after the D-luciferin injection, mice were imaged for 1–5 minutes. *In vivo* images of X-ray, luminescence (Renca-Luc), and fluorescence (Ambly750S - Ex:750 nm and Em: 790 nm), were acquired using CareStream instrument (MS FX PRO, DT, USA).

### Statistical analysis

Results were expressed as arithmetic means ± SEM. Statistical analysis was performed using the statistical analysis option in the GraphPad Prism 5.0 software (GraphPad Software Inc., San Diego, CA), unpaired *t*-tests with Welch's correction to compare the differences between Amblyomin-X treatment and untreated control. *P* < 0.05 was regarded as significantly different.

## SUPPLEMENTARY MATERIAL FIGURE


