# Supplementary material for: Promising pharmacological profile of a Kunitz-type inhibitor in murine renal cell carcinoma model
Source: Oncotarget. 2016 Aug 23;7(38):62255–66. doi: 10.18632/oncotarget.11555 (PMC5308724; doi:10.18632/oncotarget.11555)
Supplement: Supplementary file 1 [file oncotarget-07-62255-s001.pdf]

Promising pharmacological profile of a Kunitz-type inhibitor in murine renal cell carcinoma model

Supplementary Materials

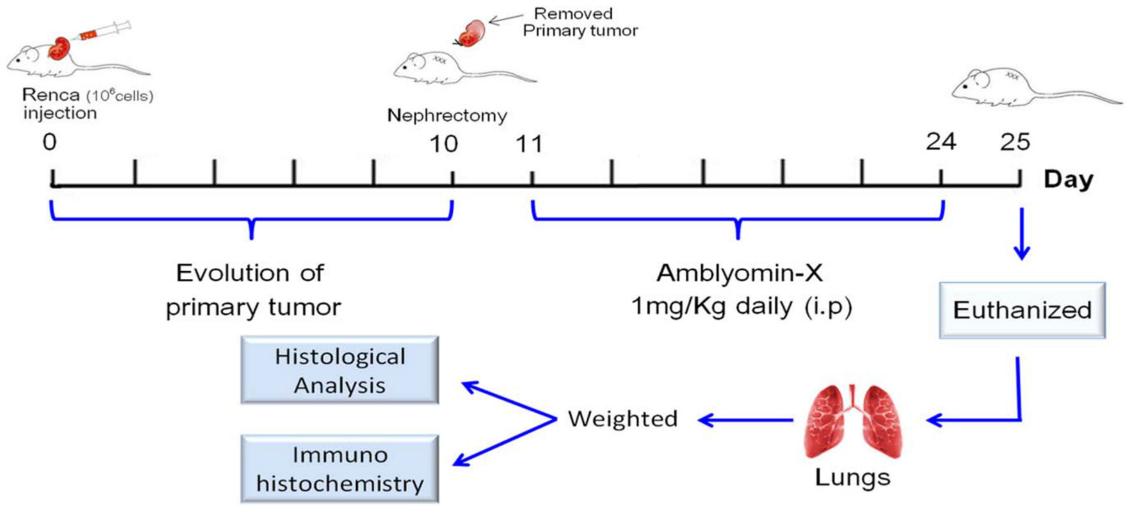

Supplementary Figure S1: Murine orthotopic kidney tumor model protocol. Renca cells (1×10^6) suspended in 50 μL of PBS were injected into the subcapsular space of the left kidney of a BALB/c mouse under anesthesia. After 10 days the kidney with the primary tumor was surgically removed. Intraperitoneal administration of saline or Amblyomin-X was started 24 h after nephrectomy and was performed daily for 14 days. Then, the animals were euthanized with deep anesthesia, followed by cardiac puncture. The lungs were collected and weighed and prepared for histological analyses or immunohistochemistry.
